# Magnetic properties of six-legged spin-1 nanotube in presence of a longitudinal applied field

**DOI:** 10.1038/s41598-019-48833-7

**Published:** 2019-08-26

**Authors:** Zakaria ElMaddahi, Moulay Youssef El Hafidi, Mohamed El Hafidi

**Affiliations:** Condensed Matter Physics Laboratory, Faculty of Science Ben M’sik, Hassan II University of Casablanca, B. P 7955, Av. D. El Harty, 20663 Casablanca, Morocco

**Keywords:** Physics, Phase transitions and critical phenomena

## Abstract

In this paper, we investigate the magnetic behavior of a single-walled hexagonal spin-1 Ising nanotube by using the effective field theory (EFT) with correlations and the differential operator technique (DOT). The system consists of six long legs distributed parallel to each other on a hexagonal basis. Within each chain, spin sites are regularly  positioned and magnetically coupled through a J_//_ exchange interaction along the chains and J⊥ between adjacent chains. Key equations of magnetization, susceptibility and critical temperatures are established, numerically resolved and carefully analyzed for some selected exchange couplings and various applied magnetic fields. In addition to the phase diagram, interesting phenomena are  noted, particularly for opposite exchange interactions where magnetization plateaus and frustration are discovered.

## Introduction

The recent decade has seen a resurgence of interest in small-sizes magnetic objects, especially at nanoscale (nanoparticles, nanotubes, nanowires, etc.). This is due, on the one hand, to advances in atomic engineering and, on the other, to their potential applications in various fields such as magnetic drug delivery^1^, bio and nanomedicine^2^, nano-magnetic resonance imagery (Nano-MRI) with nanoscale resolution^3^, permanent magnets^4^, long-lasting memories^5^ and recording media^6^.

Theoretically, the magnetic properties of nanoparticles and nanotubes characterized by their quantum and surface boundary effects^7,8^ have been widely investigated by the well-known methods of statistical and quantum physics including the effective field theory (EFT)^9,10^, Monte Carlo (MC) simulation^11^, the mean field theory (MFT)^12^, Green function formalism^13^ and Bethe-Peierls approximation^14^. For instance, Y. Kocakaplan *et al*. have studied the magnetic properties and hysteresis behaviors of a cylindrical transverse spin-1 Ising nanowire with a crystal field interaction in absence of magnetic field using the effective field theory combined with a probability distribution technique^15^. On the other hand, Wei Wang *et al*. have examined the compensation behaviors and magnetic properties of a cylindrical ferrimagnetic core-shell Ising nanotube by using MC simulation. The authors found that the system undergoes first- or second-order phase transitions for some physical parameters^16^.

Here, we aim to study the magnetic properties of a six-legged spin-1 nanotube by applying the effective-field theory (EFT) with correlations and the differential operator technique (DOT).

Results of analytical and numerical calculations for magnetization and susceptibility are presented and carefully discussed for specific values of the exchange couplings and so the external longitudinal magnetic fields. Hence, they play a key role in nanotube magnetic properties. Also, phase diagrams of the hexagonal nanotube  are  investigated. Particular attention has been given to conflicting cases of exchange couplings along and between chains constituting the nanotube. Magnetic plateaus have been revealed, reflecting the frustrations that arise in such situations.

The manuscript is structured as follows: Section 2 is destined to introduce the theoretical approach of the effective field theory (EFT) with correlations, to get expressions of magnetization, internal energy, specific heat, entropy, free energy and critical boundaries in the single-wall spin-1 Ising hexagonal nanotubes. In Section 3, we present numerical results and diagrams in a ferromagnetic and antiferromagnetic state with a special focus on frustration cases. In Section 4, we formulate some concluding remarks.

## Model and Theoretical Formulation

**Figure 1:**
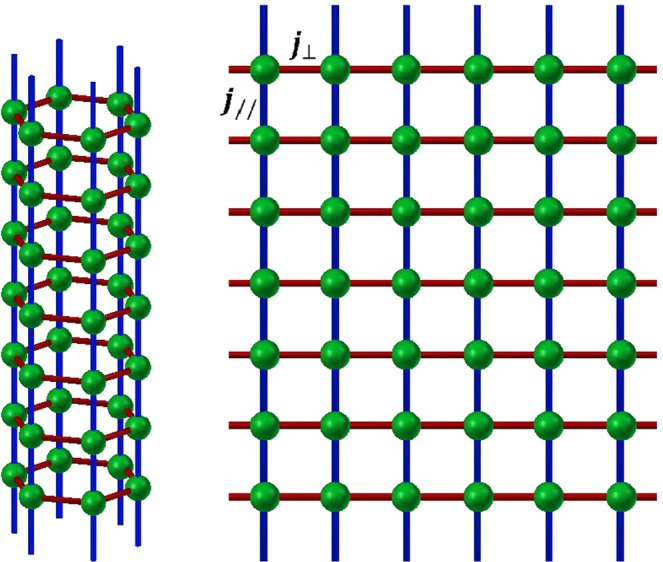
Schematic representation of the hexagonal spin nanotube. (**a**) The green spheres display the magnetic atoms (with spin S = 1). The blue and the red lines correspond respectively to the longitudinal exchange (*J*_//_) and transversal exchange (*J*_⊥_) coupling links. (**b**) The in-plane equivalent structure of the nanotube consisting on a six-chains grid with the periodic boundary condition in the horizontal direction S_i,δ+6_ ≡ S_i,δ_.

The considered model consists of a spin-1 Ising hexagonal nanotube under an external longitudinal magnetic field. The schematic stacking of the nanotube is depicted in Fig. 1. In our work, the hexagonal nanotube can be built as follows: firstly, the six chains are connected each to other forming a monolayer grid with vectors **a** and **b**, then by rolling up the monolayer along a given chiral axis **r** = n**a** + m**b**, where **a** and **b** are the in-plane lattice vectors, n and m are two integer numbers (here n = 6 and m = 0)^17^. Thus, within the obtained hexagonal nanotube, each site is occupied by a spin-1 Ising particle and each spin (i, α) on the chain α (α = 1–6) interacts not only with its two adjacent neighbors neighbor (i ± 1, α) along the chain via the longitudinal exchange J_//_ but also with its two in-plan nearest-neighbors (i, α ± 1) via the transverse component J_⊥_.

The spin system is describing by the following Hamiltonian:

1 $$H=-\,{J}_{\perp }\mathop{\sum }\limits_{i}^{N}\,\mathop{\sum }\limits_{\delta =1}^{6}\,{S}_{i,\delta }{S}_{i,\delta +1}-{J}_{//}\mathop{\sum }\limits_{i}^{N}\,\mathop{\sum }\limits_{\delta =1}^{6}\,{S}_{i,\delta }{S}_{i+1,\delta }-h\mathop{\sum }\limits_{i}^{N}\,\mathop{\sum }\limits_{\delta }^{6}\,{S}_{i,\delta }$$

where the spin operator S_z_ can take one of the three allowed eigenvalues: {±1, 0}. The two first sums run over entirely nearest neighbors pairs on the magnetic network. The last summation corresponds to the Zeeman coupling and is over all the lattice sites. J_//_ is the longitudinal exchange interaction linking two nearest-neighbor magnetic atoms along the chains and the J_⊥_ is the transverse exchange interaction acting among adjacent chains. J_r_ > 0 (r = //, ⊥) (respectively < 0) for ferromagnetic, FM (respectively antiferromagnetic, AFM). h is the applied longitudinal magnetic field.

In order to apply the EFT with correlations and the DOT for the considered spin-1 system, we reformulate the spin Hamiltonian in the simplest form:

2 $$H=-\,\mathop{\sum }\limits_{i}^{N}\,\mathop{\sum }\limits_{\delta }^{6}\,{S}_{i,\delta }{H}_{i\delta }$$

where

3 $${H}_{i\delta }=h+{J}_{\perp }\mathop{\sum }\limits_{\delta \text{'}\ne \delta }^{\,}\,{S}_{i,\delta ^{\prime} }+{J}_{//}\mathop{\sum }\limits_{i\text{'}\ne i}^{\,}\,{S}_{i^{\prime} ,\delta }$$

Thus, the configurational average of spin 〈*S*_*i*,*j*_〉 at the thermodynamic equilibrium is expressed within the framework of the EFT with correlations by^18^:

4 $$\langle {S}_{i,\delta }\rangle =\frac{1}{Z}Tr(\frac{T{r}_{i}({S}_{i,\delta }{e}^{-\beta {H}_{i\delta }})}{T{r}_{i}({e}^{-\beta {H}_{i\delta }})})\,$$

For *S*_*iδ*_ = {−1; 0; 1}

5 $$\langle {f}_{i,\delta }{S}_{i,\delta }\rangle =\langle {f}_{i}\frac{2\,sh(\beta {E}_{i\delta }+h)}{2\,ch(\beta {E}_{i\delta }+h)+1}\rangle $$

6 $$\langle {({S}_{i,\delta })}^{2}\rangle =\langle \frac{2\,ch(\beta {E}_{i\delta }+h)}{2\,ch(\beta {E}_{i\delta }+h)+1}\rangle $$

where *E*_*iδ*_ are the corresponding eigenvalues of *H*_*iδ*_.

Now, let us introduce the differential operator technique as follows

7 $$\langle {f}_{i,\delta }{S}_{i,\delta }\rangle ={\langle {f}_{i,\delta }{e}^{\nabla {E}_{i,\delta }}\rangle F(x+h)|}_{x=0}$$

where *f*_*i*,*δ*_ designs any function of spin variables except *S*_*i*,*δ*_

and

8 $$\langle {({S}_{i,\delta })}^{2}\rangle ={\langle {e}^{\nabla {E}_{i,\delta }}\rangle G(x+h)|}_{x=0}$$

where $\nabla =\frac{\partial }{\partial x}$ is a differential operator. The functions *F*(*x* + *h*) and *G*(*x* + *h*) are defined by

9 $$\langle F(x+h)\rangle =\langle \frac{2\,sh(\beta {E}_{i,\delta }+h)}{2\,ch(\beta {E}_{i,\delta }+h)+1}\rangle $$

10 $$\langle G(x+h)\rangle =\langle \frac{2\,ch(\beta \,{E}_{i\delta }+h)}{2\,ch(\beta {E}_{i\delta }+h)+1}\rangle $$

By using the identity

11 $${e}^{{\rm{\alpha }}{S}_{i,\delta }}={({S}_{i,\delta })}^{2}{\rm{ch}}({\rm{\alpha }})+{S}_{i,\delta }\,sh({\rm{\alpha }})+1-{({S}_{i,\delta })}^{2}$$

 the expression value $\langle {f}_{i,\delta }{e}^{\nabla {E}_{i,\delta }}\rangle$ reduces to

12 $$\begin{array}{rcl}\langle {f}_{i,\delta }{e}^{\nabla {E}_{i\delta }}\rangle  & = & \langle {f}_{i,\delta }\prod _{\delta ^{\prime} }[{({S}_{i,\delta ^{\prime} })}^{2}{\rm{ch}}(\nabla {J}_{\perp })+{S}_{i,\delta ^{\prime} }sh(\nabla {J}_{\perp })+1-{({S}_{i,\delta ^{\prime} })}^{2}]\\  &  & \prod _{i^{\prime} }[{({S}_{i^{\prime} ,\delta })}^{2}{\rm{ch}}(\nabla {J}_{//})+{S}_{i^{\prime} ,\delta }sh(\nabla {J}_{//})+1-{({S}_{i^{\prime} ,\delta })}^{2}]\rangle \end{array}$$

From which 〈*S*_*i*,*δ*_〉 and 〈(S_i,δ_)^2^〉 are given by (by putting *f*_*i*,*δ*_ = 1)

13 $$\begin{array}{rcl}\langle {S}_{i,\delta }\rangle  & = & \langle \prod _{\delta \text{'}}[{({S}_{i,\delta ^{\prime} })}^{2}{\rm{ch}}(\nabla {J}_{\perp })+{S}_{i,\delta ^{\prime} }sh(\nabla {J}_{\perp })+1-{({S}_{i,\delta ^{\prime} })}^{2}]\\  &  & \prod _{i\text{'}}[{({S}_{i^{\prime} ,\delta })}^{2}{\rm{ch}}(\nabla {J}_{//})+{S}_{i^{\prime} ,\delta }\,sh(\nabla {J}_{//})+1-{({S}_{i^{\prime} ,\delta })}^{2}]\rangle {F(x+\beta h)|}_{x=0}\end{array}$$

and

14 $$\begin{array}{c}\langle {({S}_{i,\delta })}^{2}\rangle =\langle \prod _{\delta \text{'}}[{({S}_{i,\delta ^{\prime} })}^{2}{\rm{ch}}(\nabla {J}_{\perp })+{S}_{i,\delta ^{\prime} }sh(\nabla {J}_{\perp })+1-{({S}_{i,\delta ^{\prime} })}^{2}]\\ \,\,\,\,\,\,\,\prod _{i\text{'}}[{({S}_{i^{\prime} ,\delta })}^{2}{\rm{ch}}(\nabla {J}_{//})+{S}_{i^{\prime} ,\delta }\,sh(\nabla {J}_{//})+1-{({S}_{i^{\prime} ,\delta })}^{2}]\rangle {G(x+\beta h)|}_{x=0}\end{array}$$

At this stage, one may evaluate the magnetization *m* = <*S*_*i*,*δ*_> for the spin-1 nanotube, by applying delicately the effective field theory (EFT) with correlations and the differential operator technique (DOT):

15 $$\begin{array}{rcl}m & = & \langle \prod _{\delta ^{\prime} }[{({S}_{i,\delta ^{\prime} })}^{2}{\rm{ch}}(\nabla {J}_{\perp })+{S}_{i,\delta ^{\prime} }\,sh(\nabla {J}_{\perp })+1-{({S}_{i,\delta ^{\prime} })}^{2}]\\  &  & \prod _{i\text{'}}[{({S}_{i^{\prime} ,\delta })}^{2}{\rm{ch}}(\nabla {J}_{//})+{S}_{i^{\prime} ,\delta }\,sh(\nabla {J}_{//})+1-{({S}_{i^{\prime} ,\delta })}^{2}]\rangle {F(x+\beta h)|}_{x=0}\end{array}$$

where $\beta =\frac{1}{{K}_{B}T}$, k_B_ being the Boltzmann constant and T the absolute temperature, $\frac{\partial }{\partial x}$ the one-dimensional differential operator which is defined by its action ${e}^{\gamma \frac{\partial }{\partial x}}f(x)=f(x+\gamma )$.

After long calculations, the quadrupolar moment *q* = 〈(*S*_*i*,*δ*_)^2^〉 measuring the mutual spins correlations within the nanotube can be expressed by the following equation:

16 $$\begin{array}{rcl}{\rm{q}} & = & \langle \prod _{\delta \text{'}}[{({S}_{i,\delta ^{\prime} })}^{2}{\rm{ch}}(\nabla {J}_{\perp })+{S}_{i,\delta ^{\prime} }\,{\rm{sh}}(\nabla {J}_{\perp })+1-{({S}_{i,\delta ^{\prime} })}^{2}]\\  &  & \prod _{i^{\prime} }[{({S}_{i^{\prime} ,\delta })}^{2}{\rm{ch}}(\nabla {{\rm{J}}}_{//})+{S}_{i^{\prime} ,\delta }\,{\rm{sh}}(\nabla {{\rm{J}}}_{//})+1-{({S}_{i^{\prime} ,\delta })}^{2}]\rangle {G({\rm{x}}+{\rm{\beta }}{\rm{h}})|}_{{\rm{x}}=0}\end{array}$$

by the use of the decoupling approximation^19^:

17 $$\langle {S}_{j}^{z}{({S}_{k}^{z})}^{2}\,\ldots \,\ldots \,\ldots \,{S}_{l}^{z}\rangle =\langle {S}_{j}^{z}\rangle \langle {({S}_{k}^{z})}^{2}\rangle \,\ldots \,\ldots \,\ldots \langle {S}_{l}^{z}\rangle $$

Thereafter, by limiting our calculations to the nearest neighbors of a given spin, the magnetization and the quadrupolar moment per site become respectively

18 $$\begin{array}{rcl}{\rm{m}} & = & \mathop{\prod }\limits_{\delta ^{\prime} =1}^{2}[{\rm{q}}\,{\rm{ch}}(\nabla {J}_{\perp })+{\rm{m}}\,{\rm{sh}}(\nabla {J}_{\perp })+1-{\rm{q}}]\\  &  & \mathop{\prod }\limits_{i^{\prime} =1}^{2}[{\rm{q}}\,{\rm{ch}}(\nabla {{\rm{J}}}_{//})+{\rm{m}}\,{\rm{sh}}(\nabla {{\rm{J}}}_{//})+1-{\rm{q}}]{{\rm{F}}({\rm{x}}+{\rm{\beta }}h)|}_{{\rm{x}}=0}\end{array}$$

19 $$\begin{array}{rcl}q & = & \mathop{\prod }\limits_{\delta ^{\prime} =1}^{2}[{\rm{q}}\,{\rm{ch}}(\nabla {J}_{\perp })+{\rm{m}}\,{\rm{sh}}(\nabla {J}_{\perp })+1-{\rm{q}}]\\  &  & \mathop{\prod }\limits_{i^{\prime} =1}^{2}[{\rm{q}}\,{\rm{ch}}(\nabla {{\rm{J}}}_{//})+{\rm{m}}\,{\rm{sh}}(\nabla {{\rm{J}}}_{//})+1-{\rm{q}}]{G(x+\beta h)|}_{x=0}\end{array}$$

Expanding the right hand side of eqs (18) and (19) and after long analytical calculations, these two keys variables can be written as

20 $$m={A}_{0}+{A}_{1}m+{A}_{2}{m}^{2}+{A}_{3}{m}^{3}+{A}_{4}{m}^{4}$$

and

21 $$q={B}_{0}+{B}_{1}m+{B}_{2}{m}^{2}+{B}_{3}{m}^{3}+{B}_{4}{m}^{4}$$

where *A*_*n*_ and *B*_*n*′_ (n, n′ = 0−4) are coefficients depending on *T*, *h*, *q*, *J*_//_ and *J*_⊥_ (their explicit expressions are given in Annex 1).

By differentiating magnetizations with respect to h, the initial susceptibility χ can be determined from the following equation:

22 $$\chi (T)={\frac{\partial m(T,h)}{\partial h}\rfloor }_{h=0}$$

Note that this technique allows, in despite of its hardness, to determine other other physical variables such as internal energy, magnetic entropy, specific heat, etc. Nevertheless, here, we restrict ourselves to the magnetization and the initial susceptibility. Numerical findings will be presented and discussed in the next section.

Let us to remember that the approach of EFT combined with the DOT is evidently accurate than the mean field approximation, nevertheless its generalization to Heisenberg-type systems, where spin interacts with its neighbors in the three directions, remains delicate and difficult to put into equation^18,19^.

## Numerical Results and Discussions

In this section, we report our analytical and numerical investigation of the magnetization, the quadrupolar moment and the magnetic susceptibility behaviors of the system. This study will allow us to characterize the order nature of transitions as well as the main interactions roles in the spin nanotube. This makes these new materials even more promising for technological applications than previously thought.

### Spontaneous magnetization

**Figure 2:**
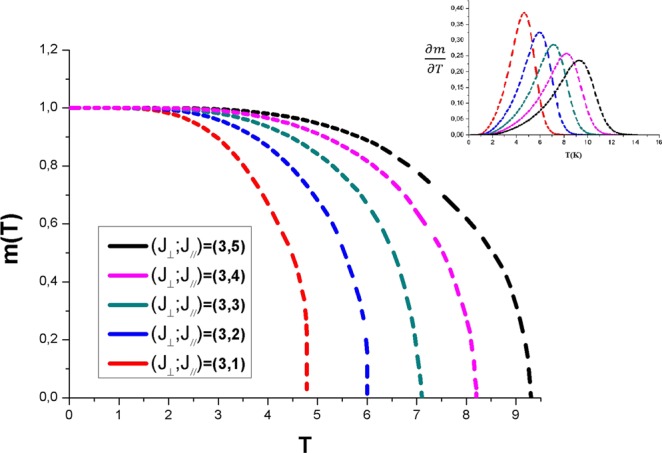
The spontaneous magnetization in Bohr magneton units as a function of the temperature for a selected value of J_⊥_ = 3 K and different values of J_//_ from 1 up to 5 K without any external field. The inset displays details of $|\frac{\partial m}{\partial T}|$ curves.

Figure 2 illustrates the thermal variation of the spontaneous magnetization obtained by solving numerically self-consistent the coupled eqs 7 and 8 for a selected set of positive transverse (J_⊥_ = 3 K) and longitudinal exchange constants (J_//_ from 1 up to 5), in the absence of the external magnetic field (h = 0). Note that h is given here in energy units.

Actually, our finding is very useful in the understanding of the ferromagnetic behavior: the spontaneous magnetizations start from the same point (m = 1) and they decrease to zero at the critical temperature T_c_ where the system displays a Ferro-Paramagnetic (FM-PM) phase transition.

Based on the graph analysis, we can conclude that the magnetization exhibits a faster decrease from the saturation value with the decreasing of exchange interactions values. However, the value of the critical temperature T_c_ increases while increasing exchange interactions (J_⊥_ and J_//_), both or one of them.

**Figure 3:**
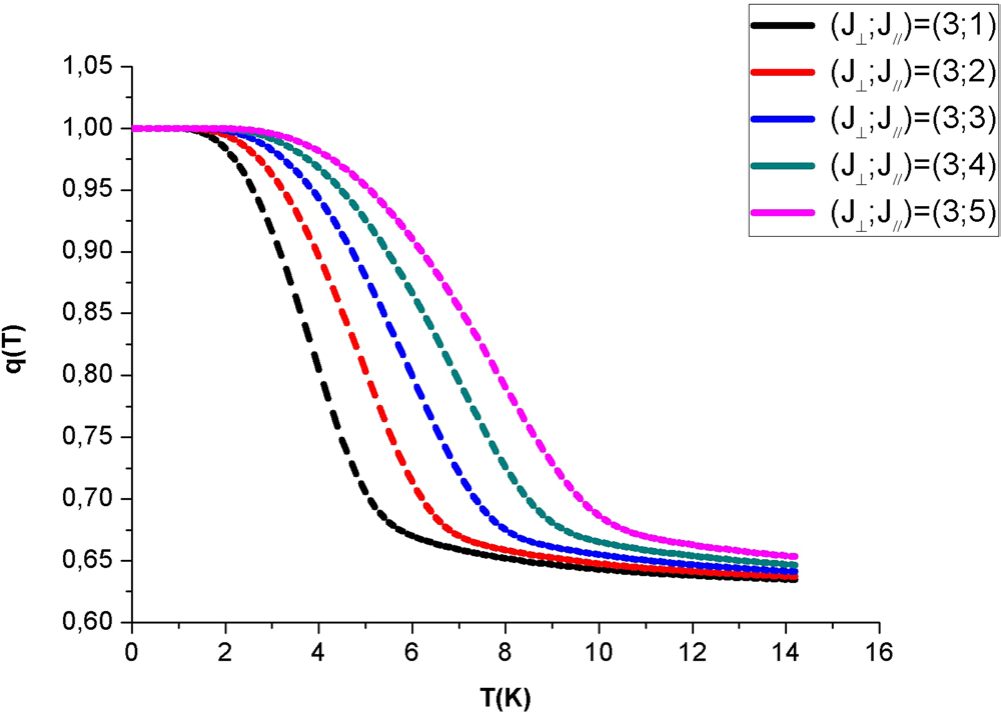
Temperature dependence of the quadrupole moment of the hexagonal nanotube for a selected value of the perpendicular exchange coupling J_⊥_ = 3 K and different values of J_//_ from 1 up to 5 K.

Figure 3  depicts the quadrupole moment as a function of temperature, for different values of longitudinal and transverse exchange parameters. We can see that the quadrupole moments starts from 1 and decrease with the increase of temperature and an inflection point at T_c_.

We note, from this figure, that typical ferromagnetic magnetization curves are obtained and that the critical temperature increases while increasing the longitudinal and/or the transversal exchange coupling.

### Magnetic susceptibility

**Figure 4:**
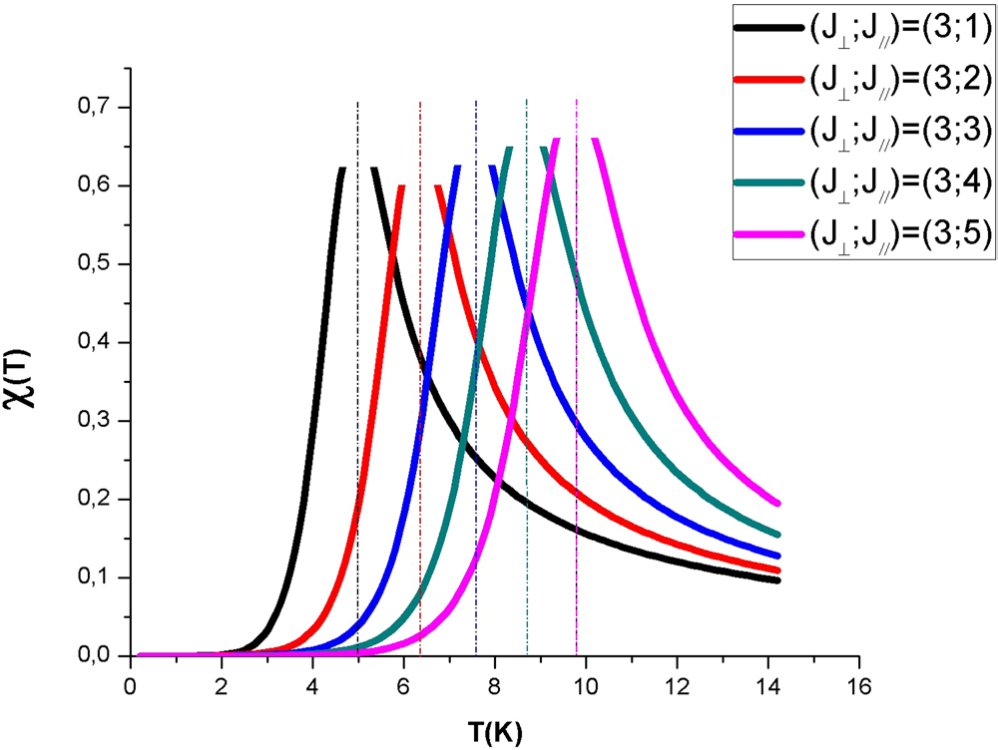
Magnetic susceptibility χ as a function of temperature T for the hexagonal nanotube with the fixed values of J_⊥_ = 3 K, J_//_ from 1 up to 5 K and h = 0. Vertical dashed lines indicate the location of the critical temperature.

Magnetic susceptibility is, in general, among tools allowing the detection and the separation between different magnetic phases thanks to the characterization of the “critical temperatures” and to the quantitative ratio between phases. Figure 4 shows the plot of magnetic susceptibility against temperature for a given value of transverse exchange constant (J_⊥_ = 3 K) and various values of longitudinal exchange interaction (J_//_ from 1 up to 5 K) without any applied field (h = 0). We notice that susceptibility increases, at first with the temperature up to a broad peak at the critical temperature for FM to PM transition and then decreases from its maximum, to weaker values with increasing temperature. This susceptibility peak is shifted to higher values when J_//_ is turned on. This is quite normal since the two exchange constants are positive corresponding to the ferromagnetism, the critical temperature increases with the increase of the exchange constant. Note that the susceptibility peak matches well with the absolute order-parameter derivative |∂*M*/∂*T*| obtained from spontaneous magnetization curves (see Fig. 2) elucidating the evidence of a second-phase order transition.

**Figure 5:**
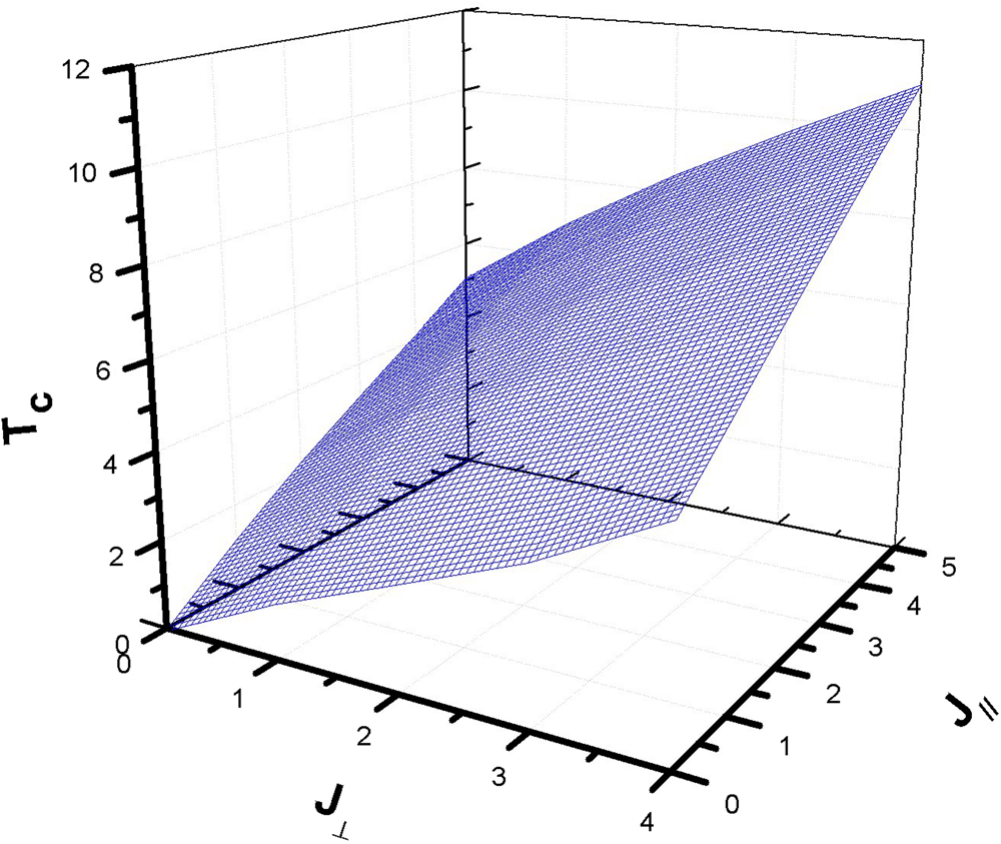
Three-dimensional plot of the phase diagram T_c_ vs (J_⊥_, J_//_) of the hexagonal spin-1 nanotube for the ferromagnetic case.

To sum up, we have drawn up in Fig. 5 a three-dimensional graph of the phase diagram for the ferromagnetic exchange case. We remark that the critical temperature is increasing monotonously with both J_⊥_ and J_//_. It is advisable to examine some standard situations, especially the case J_⊥_ = J_//_ = J = 3 K corresponding to an isotropic Ising system. We note that the value of the critical temperature found (T_c_ ≃ 7.6 K) in this case is rather close to the T_c_ corresponding to a 2D Ising system (k_B_T_c_/J = $\frac{2}{\mathrm{ln}(2+\sqrt{2)}}$ giving T_c_ ≃ 6.81 K) than to the T_c_ value established by Monte Carlo simulation for a 3D Ising system where k_B_T_c_/J ≃ 4.5 giving T_c_ ≃ 13.5 K^20^, whereas the critical temperature predicted by the mean field theory (MFT) is k_B_T_c_ = z J(S + 1)/3S ≃ 4 K^21^.

In the next section, we will look at the situation where the exchange constants are opposite in order to highlight the frustration.

### Magnetization plateaus: frustration signature

**Figure 6:**
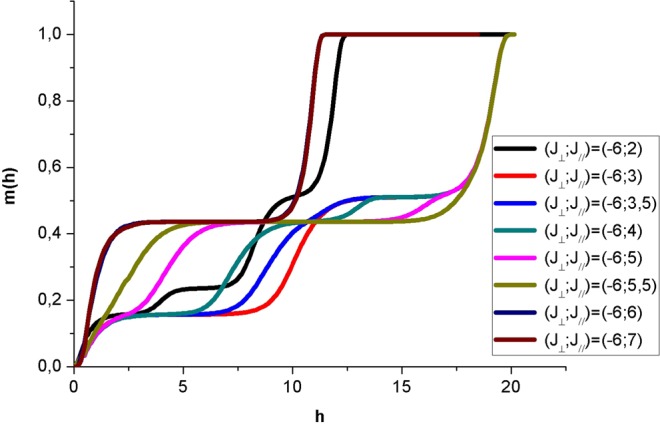
Low temperature (T = 0.40 K) isotherms of magnetization per site m of the hexagonal spin-1 nanotube for J_⊥_ = −6 K and J_//_ from 2 to 7 K (ε from 3 to 0.86). *h* is given in energy units.

It should be interesting to mention that the spin-1 hexagonal nanotube may display a rather diverse magnetization process including either one, two or three intermediate magnetization plateaus when the exchange couplings are conflicting (J_⊥_ > 0, J_//_ < 0 or J_⊥_ < 0, J_//_ > 0) giving rise to frustration. It is well known that frustration is caused by the competition of ferromagnetic and antiferromagnetic couplings, or linked to the spin lattice geometry such as in triangular antiferromagnetic structures for a review see^22^. When the frustration parameter is sufficiently small, ε = −J_⊥_/J_//_ ≤ l, one may observe just one intermediate plateau at a fraction of the saturation magnetization related to a ferrimagnetic phase due mainly to uncompensated ratios of m_s_ = +1, 0 and −1 spin states for S = 1 (see Fig. 6). When the frustration parameter is increased (for example J_⊥_ = −6, J_//_ = 4; ε = 6/4 = 1.5), a more spectacular magnetization curve with three different intermediate plateaus at 0.15, 0.45 and 0.5 of the saturation magnetization can be detected for moderate values of ε (see Fig. 6).

**Figure 7:**
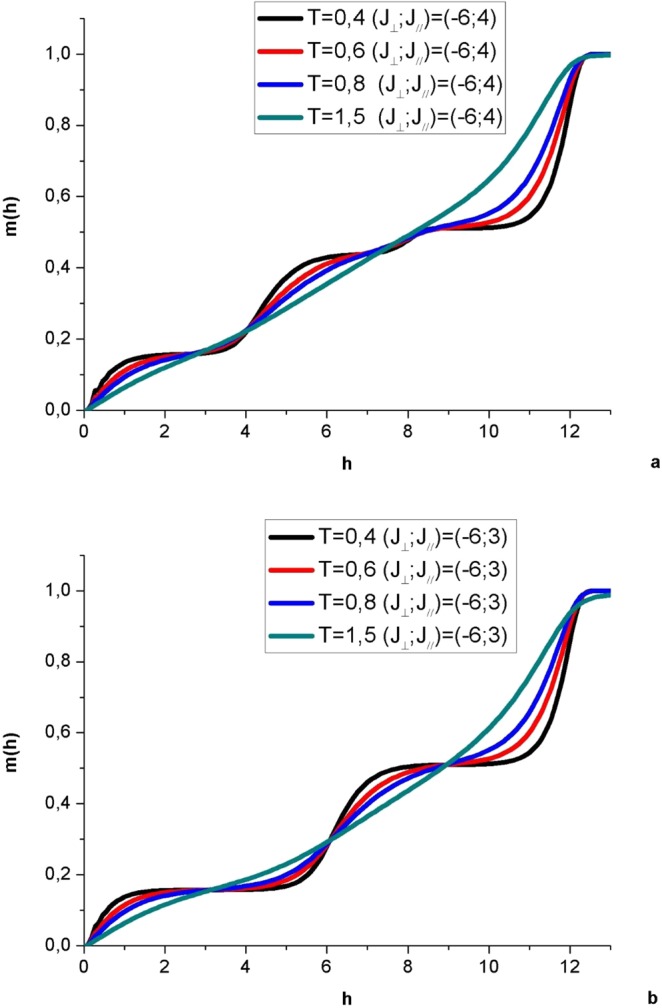
Magnetic field h dependence of magnetization m for the hexagonal nanotube with spin S = 1, J_⊥_ = −6, for two anisotropy ratio values ε = 1.5 (**a**) and ε = 2 (**b**) and T from 0.4 up to 1.5 K.

Conjointly to the magnetization plateaus observed at low temperature, sudden jumps occur at critical fields where the Zeeman contribution in the Hamiltonian (1) overcomes the frustrated exchange couplings and succeeds in tilting another proportion of spins towards their high states. This assumes the existence of energy barriers between populated levels and a residual entropy due to frustration causing quantum fluctuations. Within a plateau, the spins are confined in a highly degenerate energy band and as the field increases, the degeneracy of these levels attains its minimum degree at the saturation magnetization. At higher temperatures, the magnetization jumps begin to soften and magnetization increases gradually with the applied field showing only knees at the critical fields clearly observed at very low temperature (see Fig. 7 for ε = 1 and 1.5). These jumps tend to disappear as soon as relatively high temperatures are attained.

Experimentally, such plateaus have been observed at 1/3M_s_ and 2/3M_s_ in Cs_2_CuBr_4_^23^ and at 1/3M_s_ in Ba_3_CoSb_2_O_9_^24^ that are viewed as geometrically frustrated Heisenberg S = 1/2 systems, where quantum fluctuations may stabilize a series of spin states at simple increasing fractions of the saturation magnetization giving rise to kinks, jumps or plateaus in the magnetization curves.

## Concluding Remarks

We have investigated the thermodynamic and magnetic properties of a single-walled spin-1 hexagonal by using the effective-field theory (EFT) with correlations and the differential operator technique (DOT). Magnetization, initial susceptibility and critical boundaries were obtained. The low temperature states magnetization displays up to three intermediate plateaus at fractional values of the saturation magnetization for opposed inter- and intra- chains exchange couplings (ferro- vs antiferromagnetic couplings). Frustration can introduce ‘accidents’ in the magnetization process of this quantum system, in the form of plateaus occurring at rational values of the magnetization. At higher temperature, thermal fluctuations smoothen magnetization jumps. The special geometrical shape of the tube provides its original properties between those of the 2D and 3D systems and in the near future, this type of nanomaterials would earn a key place in various fields of applications. Finite size effects in such systems should be significant, so it would be important to consider them both analytically and by numerical simulation.

**Annex 1**

The *A*_*n*_ (n = 0–4) coefficients used in Eq. (20) are given by

$$\begin{array}{rcl}{A}_{0} & = & [1-2{q}^{4}cosh({j}_{//})cosh{({j}_{\perp })}^{2}+4{q}^{4}cosh({j}_{//})cosh({j}_{\perp })\\  &  & -\,2{q}^{4}cosh{({j}_{//})}^{2}cosh({j}_{\perp })+{q}^{4}cosh{({j}_{//})}^{2}cosh{({j}_{\perp })}^{2}\\  &  & -\,4{q}^{3}+{q}^{4}-2{q}^{4}cosh({j}_{//})+{q}^{4}cosh{({j}_{//})}^{2}+2qcosh({j}_{//})\\  &  & +\,6{q}^{3}cosh({j}_{//})-2{q}^{3}cosh{({j}_{//})}^{2}+6{q}^{2}-6{q}^{2}cosh({j}_{//})\\  &  & +\,{q}^{2}cosh{({j}_{//})}^{2}-2{q}^{4}cosh({j}_{\perp })+{q}^{4}cosh{({j}_{\perp })}^{2}+{q}^{2}cosh{({j}_{\perp })}^{2}\\  &  & +\,6{q}^{3}cosh({j}_{\perp })-2{q}^{3}cosh{({j}_{\perp })}^{2}-6{q}^{2}cosh({j}_{\perp })\\  &  & +\,2{q}^{3}cosh{({j}_{//})}^{2}cosh({j}_{\perp })-8{q}^{3}cosh({j}_{//})cosh({j}_{\perp })\\  &  & +\,2{q}^{3}cosh({j}_{//})cosh{({j}_{\perp })}^{2}+4{q}^{2}cosh({j}_{//})cosh({j}_{\perp })\\  &  & +\,2qcosh({j}_{\perp })-4q]F(x+\beta h){|}_{x=0}\end{array}$$

$$\begin{array}{rcl}{A}_{1} & = & [2sinh({j}_{\perp })+2sinh({j}_{//})-6sinh({j}_{//})q-6sinh({j}_{\perp })q-2sinh({j}_{\perp }){q}^{3}cosh{({j}_{//})}^{2}\\  &  & +\,4sinh({j}_{\perp }){q}^{3}cosh({j}_{//})+2sinh({j}_{//}){q}^{3}cosh({j}_{//})-8sinh({j}_{\perp }){q}^{2}cosh({j}_{//})\\  &  & -\,4sinh({j}_{//}){q}^{2}cosh({j}_{//})+2sinh({j}_{//})qcosh({j}_{//})+2sinh({j}_{\perp }){q}^{2}cosh{({j}_{//})}^{2}\\  &  & +\,2sinh({j}_{\perp })qcosh({j}_{\perp })-4sinh({j}_{\perp }){q}^{2}cosh({j}_{\perp })+2sinh({j}_{\perp }){q}^{3}cosh({j}_{\perp })\\  &  & +\,4sinh({j}_{//})qcosh({j}_{\perp })-8sinh({j}_{//}){q}^{2}cosh({j}_{\perp })+2sinh({j}_{//}){q}^{2}{(cosh({j}_{\perp }))}^{2}\\  &  & +\,4sinh({j}_{//}){q}^{3}cosh({j}_{\perp })-2sinh({j}_{//}){q}^{3}{(cosh({j}_{\perp }))}^{2}\\  &  & +\,4sinh({j}_{\perp })\,\ast \,{q}^{2}cosh({j}_{//})cosh({j}_{\perp })-4sinh({j}_{\perp }){q}^{3}cosh({j}_{//})cosh({j}_{\perp })\\  &  & +\,2sinh({j}_{\perp }){q}^{3}{(cosh({j}_{//}))}^{2}cosh({j}_{\perp })+4sinh({j}_{//}){q}^{2}cosh({j}_{//})cosh({j}_{\perp })\\  &  & -\,4sinh({j}_{//}){q}^{3}cosh({j}_{//})cosh({j}_{\perp })+2sinh({j}_{//}){q}^{3}cosh({j}_{//}){(cosh({j}_{\perp }))}^{2}\\  &  & -\,2sinh({j}_{\perp }){q}^{3}-2sinh({j}_{//}){q}^{3}+6sinh({j}_{//}){q}^{2}+6sinh({j}_{\perp }){q}^{2}\\  &  & +\,4sinh(j2)qcosh({j}_{//})]F{(x+\beta h)}_{x=0}\end{array}$$

$$\begin{array}{rcl}{A}_{2} & = & [sinh{({j}_{\perp })}^{2}-2sinh{({j}_{\perp })}^{2}q+2sinh{({j}_{\perp })}^{2}qcosh({j}_{//})+sinh{({j}_{\perp })}^{2}{q}^{2}\\  &  & -\,2sinh{({j}_{\perp })}^{2}{q}^{2}cosh({j}_{//})+sinh{({j}_{\perp })}^{2}{q}^{2}cosh{({j}_{//})}^{2}+4sinh({j}_{//})sinh({j}_{\perp })\\  &  & -\,8sinh({j}_{//})sinh({j}_{\perp })q+4sinh({j}_{//})sinh({j}_{\perp })\,q\,cosh({j}_{\perp })\\  &  & +\,4sinh({j}_{//})sinh({j}_{\perp }){q}^{2}-4sinh({j}_{//})sinh({j}_{\perp }){q}^{2}cosh({j}_{\perp })\\  &  & +\,4sinh({j}_{//})sinh({j}_{\perp })qcosh({j}_{//})-4sinh({j}_{//})sinh({j}_{\perp }){q}^{2}cosh({j}_{//})\\  &  & +\,4sinh({j}_{//})sinh({j}_{\perp }){q}^{2}cosh({j}_{//})cosh({j}_{\perp })+sinh{({j}_{//})}^{2}-2\,sinh{({j}_{//})}^{2}q\\  &  & +\,2sinh{({j}_{//})}^{2}qcosh({j}_{\perp })+sinh{({j}_{//})}^{2}{q}^{2}-2sinh{({j}_{//})}^{2}{q}^{2}cosh({j}_{\perp })\\  &  & +\,sinh{({j}_{//})}^{2}{q}^{2}cosh{({j}_{\perp })}^{2}]{F(x+\beta h)|}_{x=0}\end{array}$$

$$\begin{array}{rcl}{A}_{3} & = & [2sinh({j}_{//})sinh{({j}_{\perp })}^{2}-2sinh({j}_{//})sinh{({j}_{\perp })}^{2}q\\  &  & +\,2sinh({j}_{//})sinh{({j}_{\perp })}^{2}qcosh({j}_{//})+2sinh{({j}_{//})}^{2}sinh({j}_{\perp })\\  &  & -\,2sinh{({j}_{//})}^{2}sinh({j}_{\perp })q\\  &  & +\,2sinh{({j}_{//})}^{2}sinh({j}_{\perp })qcosh({j}_{\perp })]F(x+\beta h){|}_{x=0}\end{array}$$

$${A}_{4}=[sinh{({j}_{//})}^{2}sinh{({j}_{\perp })}^{2}]F(x+\beta h){|}_{x=0}$$

while the *B*_*n*′_ (n′ = 0–4) coefficients displayed in equation (21) are given as follows

$$\begin{array}{rcl}{B}_{0} & = & [1-2{q}^{4}cosh({j}_{//})cosh{({j}_{\perp })}^{2}+4{q}^{4}cosh({j}_{//})cosh({j}_{\perp })\\  &  & -\,2{q}^{4}cosh{({j}_{//})}^{2}cosh({j}_{\perp })+{q}^{4}cosh{({j}_{//})}^{2}cosh{({j}_{\perp })}^{2}\\  &  & -\,4{q}^{3}+{q}^{4}-2{q}^{4}cosh({j}_{//})+{q}^{4}cosh{({j}_{//})}^{2}+2qcosh({j}_{//})\\  &  & +\,6{q}^{3}cosh({j}_{//})-2{q}^{3}cosh{({j}_{//})}^{2}+6{q}^{2}-6{q}^{2}cosh({j}_{//})\\  &  & +\,{q}^{2}cosh{({j}_{//})}^{2}-2{q}^{4}cosh({j}_{\perp })+{q}^{4}cosh{({j}_{\perp })}^{2}+{q}^{2}cosh{({j}_{\perp })}^{2}\\  &  & +\,6{q}^{3}cosh({j}_{\perp })-2{q}^{3}cosh{({j}_{\perp })}^{2}-6{q}^{2}cosh({j}_{\perp })\\  &  & +\,2{q}^{3}cosh{({j}_{//})}^{2}cosh({j}_{\perp })-8{q}^{3}cosh({j}_{//})cosh({j}_{\perp })\\  &  & +\,2{q}^{3}cosh({j}_{//})cosh{({j}_{\perp })}^{2}+4{q}^{2}cosh({j}_{//})cosh({j}_{\perp })\\  &  & +\,2qcosh({j}_{\perp })-4q]G(x+\beta h){|}_{x=0}\end{array}$$

$$\begin{array}{rcl}{B}_{1} & = & [2sinh({j}_{\perp })+2sinh({j}_{//})-6sinh({j}_{//})q-6sinh({j}_{\perp })q-2sinh({j}_{\perp }){q}^{3}cosh{({j}_{//})}^{2}\\  &  & +\,4sinh({j}_{\perp }){q}^{3}cosh({j}_{//})+2sinh({j}_{//}){q}^{3}cosh({j}_{//})-8sinh({j}_{\perp }){q}^{2}cosh({j}_{//})\\  &  & -\,4sinh({j}_{//}){q}^{2}cosh({j}_{//})+2sinh({j}_{//})qcosh({j}_{//})+2sinh({j}_{\perp }){q}^{2}cosh{({j}_{//})}^{2}\\  &  & +\,2sinh({j}_{\perp })qcosh({j}_{\perp })-4sinh({j}_{\perp }){q}^{2}cosh({j}_{\perp })\\  &  & +\,2sinh({j}_{\perp }){q}^{3}cosh({j}_{\perp })+4sinh({j}_{//})qcosh({j}_{\perp })-8sinh({j}_{//}){q}^{2}cosh({j}_{\perp })\\  &  & +\,2sinh({j}_{//}){q}^{2}{(cosh({j}_{\perp }))}^{2}+4sinh({j}_{//}){q}^{3}cosh({j}_{\perp })\\  &  & -\,2sinh({j}_{//}){q}^{3}{(cosh({j}_{\perp }))}^{2}+4sinh({j}_{\perp })\,\ast \,{q}^{2}cosh({j}_{//})cosh({j}_{\perp })\\  &  & -\,4sinh({j}_{\perp }){q}^{3}cosh({j}_{//})cosh({j}_{\perp })+2sinh({j}_{\perp }){q}^{3}{(cosh({j}_{//}))}^{2}cosh({j}_{\perp })\\  &  & +\,4sinh({j}_{//}){q}^{2}cosh({j}_{//})cosh({j}_{\perp })-4sinh({j}_{//}){q}^{3}cosh({j}_{//})cosh({j}_{\perp })\\  &  & +\,2sinh({j}_{//}){q}^{3}cosh({j}_{//}){(cosh({j}_{\perp }))}^{2}-2sinh({j}_{\perp }){q}^{3}-2sinh({j}_{//}){q}^{3}\\  &  & +\,6sinh({j}_{//}){q}^{2}+6sinh({j}_{\perp }){q}^{2}+4sinh(j2)qcosh({j}_{//})]{G(x+\beta h)|}_{x=0}\end{array}$$

$$\begin{array}{rcl}{B}_{2} & = & [sinh{({j}_{\perp })}^{2}-2sinh{({j}_{\perp })}^{2}q+2sinh{({j}_{\perp })}^{2}qcosh({j}_{//})+sinh{({j}_{\perp })}^{2}{q}^{2}\\  &  & -\,2sinh{({j}_{\perp })}^{2}{q}^{2}cosh({j}_{//})+sinh{({j}_{\perp })}^{2}{q}^{2}cosh{({j}_{//})}^{2}+4sinh({j}_{//})sinh({j}_{\perp })\\  &  & -\,8sinh({j}_{//})sinh({j}_{\perp })q+4sinh({j}_{//})\,sinh({j}_{\perp })\,q\,cosh({j}_{\perp })\\  &  & +\,4sinh({j}_{//})sinh({j}_{\perp }){q}^{2}-4sinh({j}_{//})sinh({j}_{\perp }){q}^{2}cosh({j}_{\perp })\\  &  & +\,4sinh({j}_{//})sinh({j}_{\perp })qcosh({j}_{//})-4sinh({j}_{//})sinh({j}_{\perp }){q}^{2}cosh({j}_{//})\\  &  & +\,4sinh({j}_{//})sinh({j}_{\perp }){q}^{2}cosh({j}_{//})cosh({j}_{\perp })+sinh{({j}_{//})}^{2}\\  &  & -\,2\,sinh{({j}_{//})}^{2}q+2sinh{({j}_{//})}^{2}qcosh({j}_{\perp })+sinh{({j}_{//})}^{2}{q}^{2}\\  &  & -\,2sinh{({j}_{//})}^{2}{q}^{2}cosh({j}_{\perp })+sinh{({j}_{//})}^{2}{q}^{2}cosh{({j}_{\perp })}^{2}]{G(x+\beta h)|}_{x=0}\end{array}$$

$$\begin{array}{rcl}{B}_{3} & = & [2sinh({j}_{//})sinh{({j}_{\perp })}^{2}-2sinh({j}_{//})sinh{({j}_{\perp })}^{2}q\\  &  & +\,2sinh({j}_{//})sinh{({j}_{\perp })}^{2}qcosh({j}_{//})+2sinh{({j}_{//})}^{2}sinh({j}_{\perp })\\  &  & -\,2sinh{({j}_{//})}^{2}sinh({j}_{\perp })q\\  &  & +\,2sinh{({j}_{//})}^{2}sinh({j}_{\perp })qcosh({j}_{\perp })]G(x+\beta h){|}_{x=0}\end{array}$$

$${B}_{4}=[sinh{({j}_{//})}^{2}sinh{({j}_{\perp })}^{2}]G(x+\beta h){|}_{x=0}$$
